# CRISPR/Cas9-mediated point mutations improve α-amylase secretion in *Saccharomyces cerevisiae*

**DOI:** 10.1093/femsyr/foac033

**Published:** 2022-07-01

**Authors:** Yanyan Wang, Xiaowei Li, Xin Chen, Verena Siewers

**Affiliations:** Department of Biology and Biological Engineering, Chalmers University of Technology, Kemivägen 10, SE-41296 Gothenburg, Sweden; Department of Biology and Biological Engineering, Chalmers University of Technology, Kemivägen 10, SE-41296 Gothenburg, Sweden; Department of Biology and Biological Engineering, Chalmers University of Technology, Kemivägen 10, SE-41296 Gothenburg, Sweden; Novo Nordisk Foundation Center for Biosustainability, Chalmers University of Technology, Kemivägen 10, SE-41296 Gothenburg, Sweden; Department of Biology and Biological Engineering, Chalmers University of Technology, Kemivägen 10, SE-41296 Gothenburg, Sweden; Novo Nordisk Foundation Center for Biosustainability, Chalmers University of Technology, Kemivägen 10, SE-41296 Gothenburg, Sweden

**Keywords:** recombinant proteins, CRISPR/Cas9, point mutation, gene deletion, protein production

## Abstract

The rapid expansion of the application of pharmaceutical proteins and industrial enzymes requires robust microbial workhorses for high protein production. The budding yeast *Saccharomyces cerevisiae* is an attractive cell factory due to its ability to perform eukaryotic post-translational modifications and to secrete proteins. Many strategies have been used to engineer yeast platform strains for higher protein secretion capacity. Herein, we investigated a line of strains that have previously been selected after UV random mutagenesis for improved α-amylase secretion. A total of 42 amino acid altering point mutations identified in this strain line were reintroduced into the parental strain AAC to study their individual effects on protein secretion. These point mutations included missense mutations (amino acid substitution), nonsense mutations (stop codon generation), and frameshift mutations. For comparison, single gene deletions for the corresponding target genes were also performed in this study. A total of 11 point mutations and seven gene deletions were found to effectively improve α-amylase secretion. These targets were involved in several bioprocesses, including cellular stresses, protein degradation, transportation, mRNA processing and export, DNA replication, and repair, which indicates that the improved protein secretion capacity in the evolved strains is the result of the interaction of multiple intracellular processes. Our findings will contribute to the construction of novel cell factories for recombinant protein secretion.

## Teaser

Systematic characterization of point mutations from evolved strains using CRISPR/Cas9 technology revealed a set of gene alterations that improved recombinant protein secretion in *Saccharomyces cerevisiae*.

## Introduction

Since the biosynthesis of the first biopharmaceutical human insulin in 1982, recombinant proteins have become a mainstay of the biotechnological and biopharmaceutical industry (Anné *et al*. 2012). To meet the sharply increasing demand of recombinant proteins, it is necessary to exploit efficient expression systems ensuring high protein productivity. Over the years, several production hosts have been established ranging from bacteria to mammalian cells (Rosano and Ceccarelli 2014, Vieira Gomes *et al*. 2018). Among them, yeasts have been widely applied for industrial-scale recombinant protein production (Mattanovich *et al*. 2012, Nielsen 2013, Jozala *et al*. 2016). Baker’s yeast *Saccharomyces cerevisiae* often serves as a cell factory due to the easy genetic manipulation, the capacity of post-translational modifications, and more importantly, a well-studied protein secretion pathway (Liu *et al*. 2013, Vieira Gomes *et al*. 2018). However, heterologous protein secretion of *S. cerevisiae* is not optimal due to the low efficiency of its native secretory machinery, accompanied by frequently observed intracellular protein retention, as well as incorrect protein folding or nondesired post-translational modifications (Thak *et al*. 2020). A large body of research has, therefore, focused on engineering the protein secretory pathway, such as engineering translocation into the endoplasmic reticulum (ER; Besada-Lombana and Da Silva 2019), protein folding (Valkonen *et al*. 2003, de Ruijter *et al*. 2016), vesicle formation and trafficking (Kanjou *et al*. 2007, Hou *et al*. 2012) and ER-associated degradation (ERAD; Idiris *et al*. 2010, Piirainen and Frey 2020). The most frequently used genetic engineering strategies are deletion and overexpression of related constituent genes. Yet, the protein secretory pathway is linked to a complex structural and regulatory network within the cell, which limits rational metabolic engineering for generating optimal phenotypes. Thus, it is desirable to identify new targets for developing optimal yeast platform strains with high-level protein production.

In previous studies, *S. cerevisiae* mutant libraries with significantly increased secretion of heterologous protein α-amylase were obtained through repeated rounds of UV random mutagenesis (Liu *et al*. 2014, Huang *et al*. 2015). A total of nine clones with significantly improved α-amylase production were isolated by two different screening approaches, including manual screening (Liu *et al*. 2014) and high-throughput droplet microfluidic screening (Huang *et al*. 2015). After whole-genome sequencing of the parental strain AAC and the nine mutant strains, 146 amino acid sequence altering mutated genes were identified (Huang *et al*. 2015). However, only a few genes were chosen to evaluate their impact on protein production through either single gene-deletion or single gene-overexpression (Huang *et al*. 2017, 2018). Due to the multiple possible effects of amino acid substitutions, such as enhancing or reducing the protein activity, inactivating the whole protein function, gene deletion, and overexpression might not be ideal to reveal the connection between the mutated genes and the improved protein production (Thak *et al*. 2020). Instead, the point mutations might have resulted in a fine-tuning of gene expression level or protein activity leading to the observed effect(s) (Wang *et al*. 2019). For example, an amino acid substitution of aspartic acid with glycine in position 118 of Mpc1p involved in mitochondrial pyruvate transmembrane transport provided resistance to UK-5099, a specific inhibitor of mitochondrial pyruvate carrier activity, while the deletion of *MPC1* led to a more severe deficiency in pyruvate uptake compared to a strain containing the wild-type *MPC1* (Bricker *et al*. 2012). To explore potentially beneficial gene targets and help understand the regulatory system involved in improved protein production, it is, therefore, essential to introduce the exact point mutations when following a reverse engineering approach.

**Figure 1.**
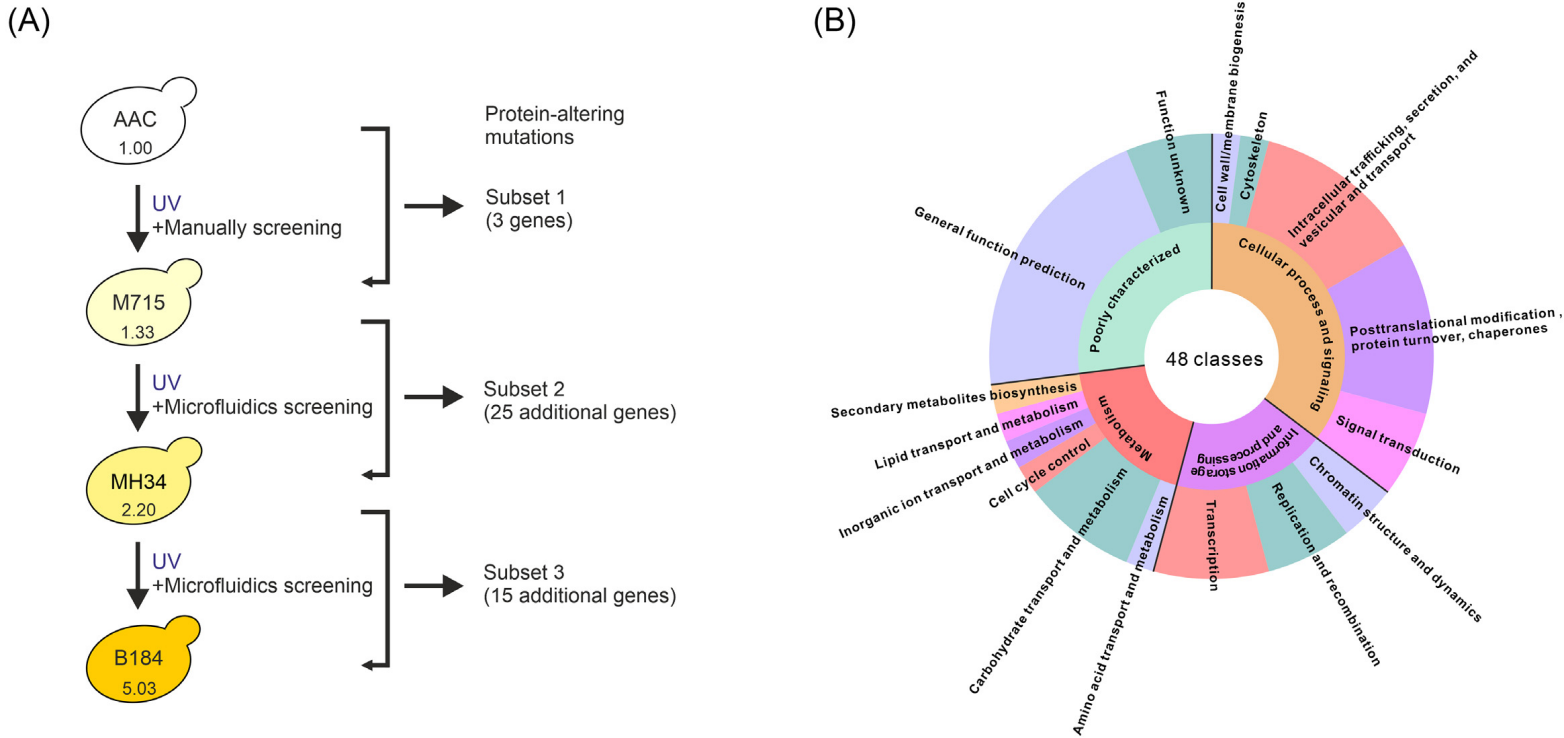
The distribution of protein-altering mutations in the *S. cerevisiae* strains evolved toward improved α-amylase production. (A) Evolutionary relationships of the mutant strains. The mutant strains were generated through repeated UV mutagenesis and two different screening approaches, including manual screening (Liu *et al*. 2014) and high-throughput microfluidics screening (Huang *et al*. 2015). Compared with the parental strain AAC, M715 showed a 1.33-fold increase, its descendant MH34 a 2.20-fold increase, and the best producer strain B184 a 5.03-fold increase in α-amylase titer in batch cultures, respectively (Huang *et al*. 2017). The number of genes with protein-sequence altering mutations after each round of mutagenesis and selection is indicated on the right. (B) Functional enrichment of mutated genes based on KOG analysis. The 43 genes are divided into 48 classes because *TIR3*, *EDE1*, *BIK1*, *TOM1*, and *ELP4* each correspond to two classes, and these mutations mainly belong to 16 classes from four groups. The middle arc represents the KOG group. The outer arc represents the KOG class.

Here, we investigated the potential targets in the evolutionary line leading from the parental strain AAC to the best protein producer B184 (Fig. 1A). We used clustered regularly interspaced short palindromic repeats (CRISPR)/CRISPR-associated protein 9 (Cas9)-mediated point mutation for testing the effect of 42 amino acid altering genes. Notably, a point mutation can change protein activity but can also abolish protein function. It will be difficult to identify the detailed mechanism if only a point mutation is performed. Therefore, whole-gene deletion was performed as a comparison in this study. The popular industrial enzyme, α-amylase, was used to assess the protein secretion. Targets identified here not only help to understand factors involved in protein secretion in yeast, but also provide a foundation for engineering other platform microorganisms.

## Results and discussion

### Classification of 43 genes mutated in evolved strains

In the previous study, through ultraviolet mutagenesis, mutants with significantly improved protein production were isolated from strain M715 (selected via manual screening) and eight additional mutant strains (selected via droplet microfluidics screening; Liu *et al*. 2014, Huang *et al*. 2015). B184 showed the best performance in α-amylase production among these mutant strains and its ancestral strains are MH34, M715, and AAC, respectively (Fig. 1A; Huang *et al*. 2015). In this evolutionary line from AAC to B184, in total 43 amino acid sequence altering mutated genes had been identified through whole genome sequencing (Huang *et al*. 2015). These mutations were divided into three subsets representing each evolutionary step: subset 1 (AAC strain to M715 strain) contained three missense mutations, subset 2 (M715 strain to MH34 strain) contained 21 missense mutations, three nonsense mutations, and one frameshift mutation, and subset 3 (MH34 strain to B184 strain) contained 14 missense mutations and one nonsense mutation (Fig. 1A; Table S1, Supporting Information). Subsequently, a functional enrichment analysis was performed according to the euKaryotic Orthologous Groups (KOG) database (Tatusov *et al*. 2003). These 43 genes could be classified into four groups and 16 subclasses (Fig. 1B). Most of the subclasses were previously found significantly upregulated in the transcriptome of B184 strain, compared with the parental strain AAC (Huang *et al*. 2017). The top four enriched subclasses were (1) intracellular trafficking, secretion, and vesicular transport; (2) post-translational modification, protein turnover, and chaperones (mainly ubiquitination); (3) transcription; and (4) carbohydrate transport and metabolism (Fig. 1B). This distribution is consistent with the previous study, which showed that the production of α-amylase results in oxidative stress in the ER, causing the activation of unfolded protein response (UPR) and the enhancement of protein folding capacity to release oxidative stress through the inhibition of the protein synthesis pathway and the improvement of the secretion pathway (Huang *et al*. 2017).

In order to better understand the driving forces during each of the three evolutionary steps, we decided to reverse engineer the parental strain with these 43 genes. Supplementarily, we also performed gene deletions to further explore the potential effect of the mutations. Among the 43 genes, *BIK1* is located on chromosome III, and this chromosome is duplicated in both MH34 and B184 (Huang *et al*. 2017). In addition, previous transcriptome analysis demonstrated that most genes on chromosome III are upregulated in the evolved strains (Huang *et al*. 2017). Considering that the effect of *BIK1* on protein production most likely resulted from its enhanced expression level, we did not include it in this work.

**Figure 2.**
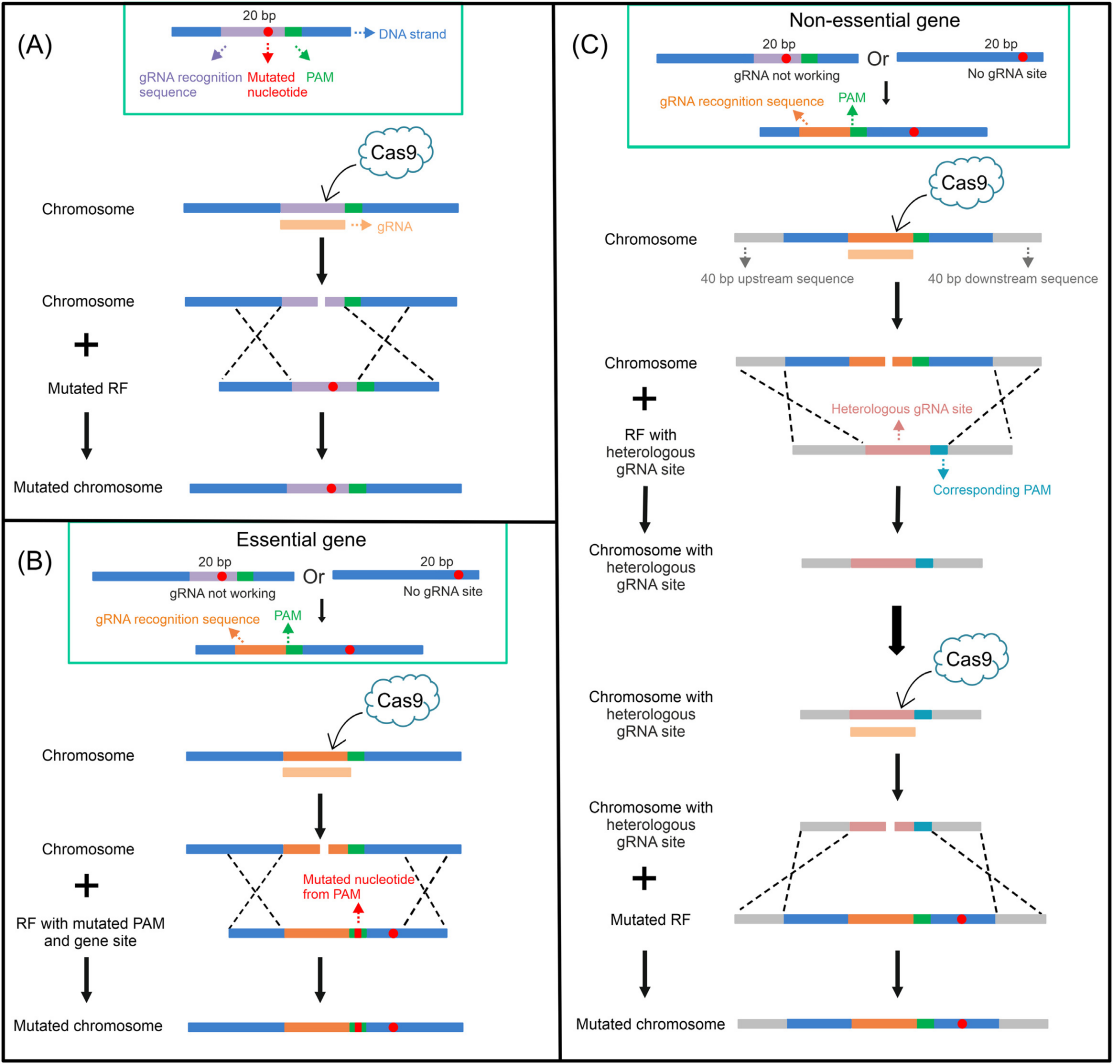
Schematic diagram of the three strategies for the introduction of the desired point mutations by CRISPR/Cas9. The repair fragments containing the point mutation or the heterologous gRNA recognition site are introduced into the chromosome of the parental strain. An all-in-one plasmid with a *kanMX* marker, a high-fidelity *cas9* nuclease gene, and the respective gRNA gene was used for genome editing. RF, repair fragment. (A) Strategy 1 was applied when the mutated site was located within the gRNA recognition sequence (20 bp) and the corresponding gRNA worked well. (B) Strategy 2 was applied when the gRNA selected for scenario (A) did not work or the mutated site was not located within the gRNA recognition sequence and the gene was essential. (C) Strategy 3 was applied when the gRNA selected for scenario (A) did not work or the mutated site was not located within the gRNA recognition sequence and the gene was nonessential.

The single or multiple-nucleotide point mutations for all the 42 gene targets were introduced into the parental strain AAC, expressing α-amylase from the plasmid CPOTud (Liu *et al*. 2012), using the CRISPR/Cas9 system. For genes containing multiple-nucleotide point mutations, multiple mutation sites were introduced simultaneously. In this work, three allele replacement strategies were designed to introduce these amino acid-altering mutations. In the first strategy, DNA double-strand breaks (DSBs) were induced in close proximity to the intended mutation, and the substituted base pair was introduced with a repair fragment so that this also altered the target sequence to avoid the persistent activity of the Cas9 nuclease (Fig. 2A). The second strategy was designed for essential genes, when strategy 1 was not possible. The repair fragment contained both the desired mutation and a mutation in the protospacer adjacent motif (PAM) that would however not alter the protein sequence (Fig. 2B). The third strategy was applied for manipulating nonessential genes, for which application of strategy 1 was not possible, and consisted of two steps (Fig. 2C). First, a heterologous gRNA recognition sequence and its PAM sequence were introduced to replace the original DNA sequence. Then, the gRNA corresponding to the heterologous sequence was expressed directing Cas9 nuclease to cleave the genomic sequence. The new DSB was repaired using the DNA sequence of the specific gene with the point mutation included. The strategies for gene deletion are illustrated in Figure S1A (Supporting Information) and Fig. 2(C) (first step).

For the first strategy, strain K01 was applied as the parental strain with Cas9 expressed from a high-copy plasmid under control of the *TEF1* promoter. Using the second strategy, four essential genes including *TFC4*, *RSP5*, *USO1*, and *CWC2* were mutated, but not subjected to the gene deletion as their deleted genotypes are not viable. When the heterologous gRNA recognition sequence in strain K01 was replaced with the repair fragment containing the desired point mutations (Fig. 2C), we observed a low efficiency with only 4 out of 19 colonies showing the desired mutation for the gene *BPT1* (Figure S2A, Supporting Information). To increase the cutting efficiency of Cas9 nuclease, the cassette encoding the Cas9 protein together with its promoter and terminator (*TEF1p*-*cas9*-*CYC1t*) was integrated into the chromosome XI-3 locus in strain K01, generating strain KC01 (Figure S1B, Supporting Information). The introduction of the *cas9* gene into the genome had no significant effect on α-amylase production and cell growth compared to strain K01 (Figure S2C and S2D, Supporting Information). The KC01 strain was then applied as parental strain for strategies 2 and 3 in later experiments. When the mutated repair fragment for *BPT1* was introduced into the strain KC01 together with the gRNA plasmid, the number of correct colonies improved from 4/19 to 12/12 (Figure S2B, Supporting Information). The strategies and strains for the introduction of each point mutation and gene deletion are listed in Table S2 (Supporting Information).

### Mutation of CBP2 increases α-amylase production

**Figure 3.**
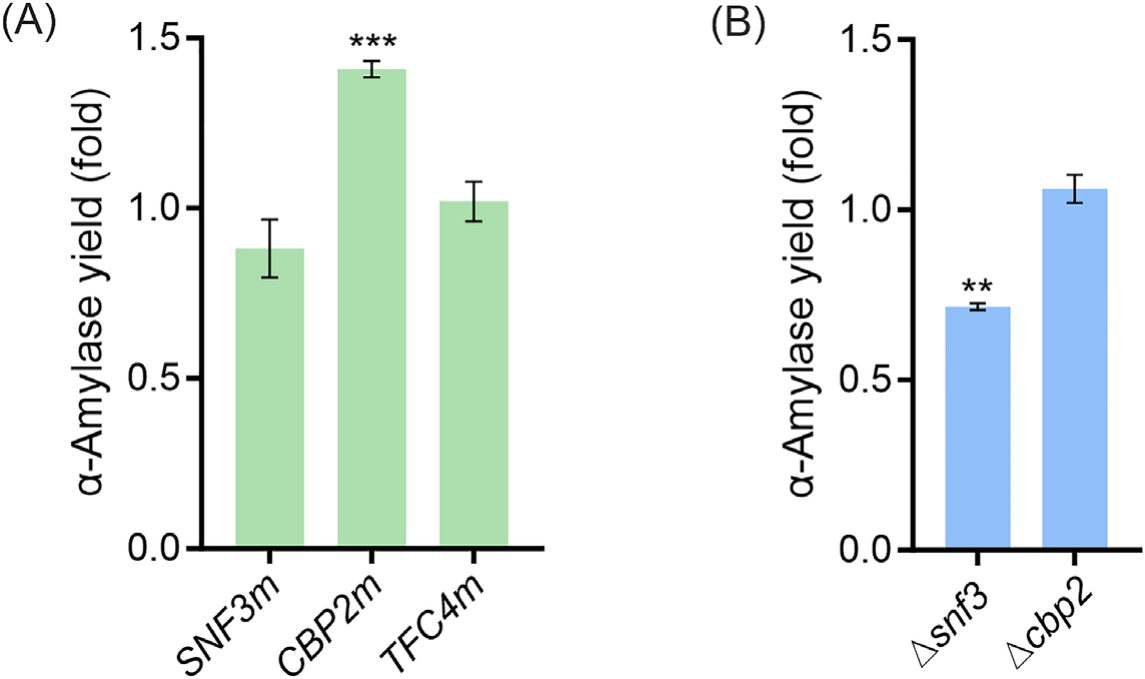
α-Amylase production after introduction of point mutations (A) or gene deletions (B) of genes identified as mutated in strain M715 (subset 1). Supernatants were collected after 96 h of cultivation in SD-2xSCAA medium at 30°C. α-Amylase activity was determined using an enzymatic kit, the parental strains K01 or KC01 were used as controls. The *Y*-axis represents the fold change of α-amylase activity per OD_600_ (yield) compared to the control strain. Data shown are average values ± SD of independent biological triplicates. Statistical significance was analyzed using a two-tailed Student’s *t*-test (**P < .01 and ***P < .001).

To evaluate the individual contribution of the three candidate mutations identified in strain M715 on α-amylase secretion (Fig. 1A; Table S1, Supporting Information), we performed single-nucleotide point mutations of *SNF3*(S97F), *CBP2*(M385T), and *TFC4*(L273F) as well as single gene deletion of *SNF3* and *CBP2* as control. *TFC4* was not deleted since it is an essential gene (Marck *et al*. 1993). The α-amylase production level in SD-2xSCAA medium after 96 h of cultivation varied in the strains with the different gene modifications (Fig. 3; Figure S3C, Supporting Information). Besides the production levels, the final cell biomass was also affected by the gene mutations and deletions (Figure S3A and S3B, Supporting Information). Out of these five strains, only the point mutation of *CBP2* improved α-amylase production 1.41-fold compared with the control strain K01 (Fig. 3A). The deletion of *CBP2* had a significantly negative effect on cell growth and greatly reduced the protein production level (Figure S3B and S3C, Supporting Information). The genetic modifications of *SNF3* and *TFC4* did not significantly increase the protein production. Deletion of *SNF3* even reduced the α-amylase level (Fig. 3B).

Cbp2p, a nuclear-encoded protein, is involved in mitochondrial mRNA processing and is required for the excision of a single catalytically active intervening sequence from the terminal intron of cytochrome b (*COB*) pre-mRNA (Gampel *et al*. 1989, Gampel and Cech 1991). Cobp is one of the catalytic subunits of the ubiquinol–cytochrome c reductase complex. This complex is a conserved component of the mitochondrial respiratory chain and essential to the process of oxidative phosphorylation (Hunte *et al*. 2003). As the splicing of the fifth intron of *COB* requires Cbp2p, the deletion of *CBP2* can affect the expression of the mitochondrial COB gene and thus block the electron transport chain in the mitochondria. The deficient respiration will in turn cause reduced cell growth or no growth. This is consistent with our result, as the deletion of *CBP2* resulted in lower biomass and protein production levels (Figure S3B and S3C, Supporting Information). This led us to speculate that the point mutation of *CBP2* might only partially impair the function of Cobp and, therefore, result in a reduced respiration. A previous study also demonstrated that anaerobic/hypoxic conditions were favorable for protein secretion (Huang *et al*. 2017), which might be the reason why a potentially respiration-impaired *CBP2* mutant strain exhibited improved α-amylase production. Indeed, under oxygen-limiting conditions, the control strain K01 was able to generate 1.59-fold more α-amylase compared to aerobic cultivation conditions (Figure S4, Supporting Information).

### The modification of six genes in subset 2 has a positive effect on α-amylase production

**Figure 4.**
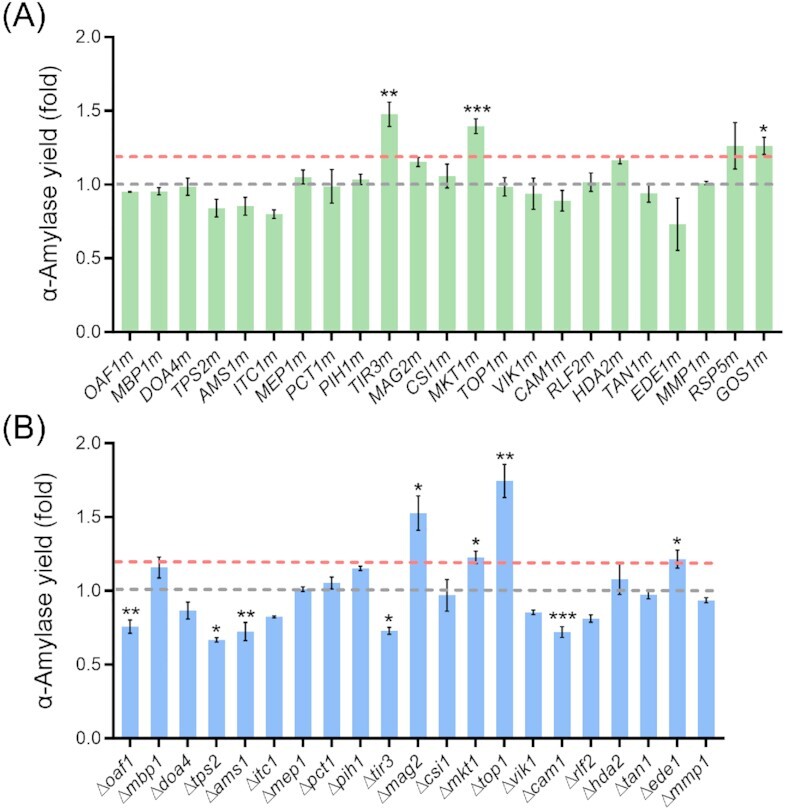
α-Amylase production after introduction of point mutations (A) or gene deletions (B) of genes identified as additionally mutated in strain MH34 (subset 2). Supernatants were collected after 96 h of cultivation in SD-2xSCAA medium at 30°C and the parental strains K01 or KC01 were used as controls. The dotted gray line indicates the α-amylase production of the control strain for comparison. The dotted pink line indicates a 1.2-fold increase in α-amylase production compared to the control strain. The *Y*-axis represents the fold change of α-amylase activity per OD_600_ (yield) compared to the control strain. Data shown are average values ± SD of independent biological triplicates. The statistical significance was listed when the α-amylase yield change was more than 1.2-fold or less than 0.8-fold compared to the control (*P < .05, **P < .01, and ***P < .001).

To verify whether the 25 gene alterations identified in strain MH34 (subset 2) had a positive effect on α-amylase production, except for *BIK1*, 24 genes were subject to single- or multiple-nucleotide point mutations and 22 genes were individually deleted except for essential genes *RSP5* (Sangkaew *et al*. 2022) and *USO1* (Heo *et al*. 2020). For *USO1*, we did not obtain any mutated clones with two different gRNAs tested. Besides, we did not evaluate the *GOS1* deletion strain, as this modification severely reduced cell growth. After obtaining the colonies carrying either point mutations or gene deletions, the α-amylase production capacity was evaluated. A total of eight out of the 23 point mutations significantly changed the α-amylase yield, with five significantly increasing α-amylase production and three significantly decreasing α-amylase production (*P* < .05) compared to the control strain (Fig. 4A). A total of 13 out of the 22 single gene deletions significantly changed the α-amylase yield, with five increasing α-amylase production and eight decreasing α-amylase production (*P* < .05) compared to the control strain (Fig. 4B). Figure S5 (Supporting Information) illustrates the biomass changes compared with the control strain. To explore these genes in more depth, the targets for which the modification led to a significant enhancement in protein yield by more than 1.2-fold were chosen for further discussion. In total, six genes were selected. For the genes *TIR3* and *GOS1*, only the point mutations improved the α-amylase yield, while the deletion of *TIR3* significantly decreased protein production (*P* < .05). For three genes, *MAG2*, *TOP1*, and *EDE1*, only the gene deletions significantly increased the protein production. For *MKT1*, both point mutation and gene deletion were shown to improve protein production. The α-amylase production yield after modification of these six genes was increased between 1.21- and 1.75-fold compared to the control strain (Fig. 4).

Tir3p, a cell wall mannoprotein, is expressed under anaerobic/hypoxic conditions (Abe 2007, Tran Nguyen Hoang *et al*. 2018). Our previous study demonstrated that the expression of *TIR3* and several other genes related to anaerobic metabolism was significantly upregulated in all evolved strains (Huang *et al*. 2017), indicating that the changes otherwise characteristic of anaerobically grown strains that were seen in the modified strains are beneficial for protein secretion. In connection with the fact that *TIR3* deletion decreased protein secretion (Fig. 4B), the potential mechanism for the positive effect of the *TIR3* point mutation on protein secretion might be due to an increase in Tir3p activity.

While the precise function of Mkt1p has not been identified, this protein shows similarity to nucleases. It was proved that Mkt1p functions as a positive post-transcriptional regulator of the *HO* gene involved in mating type switching (Tadauchi *et al*. 2004). The inactivation of *MKT1* previously decreased the formation frequency of petite colonies during serial passages and compromised the growth of these petite cells (Dimitrov *et al*. 2009). We speculate that loss of function of *MKT1* resulting in higher cell viability presumably benefits protein accumulation. *MKT1* appears to play an important role in diverse cellular functions under stress. A previous study showed that both deletion of *MKT1* and expression of the S288c allele of *MKT1* could modulate a small fraction of *TPS1* (involved in trehalose biosynthesis and cellular stresses responses) deletion cells to enter the persister-like state that enables survival under stress (Gibney *et al*. 2020). The S288c allele of *MKT1* also led to higher sensitivity to some genotoxic agents such as 4-nitroquinoline 1-oxide (Demogines *et al*. 2008, Kim and Fay 2009). Furthermore, the loss of function allele of *MKT1* is responsible for higher sporulation efficiency and ethanol tolerance (Deutschbauer and Davis 2005, Swinnen *et al*. 2012). Thus, *MKT1* has been associated with a variety of stress-related phenotypes, but not with protein production or folding stress yet.

Top1p encodes topoisomerase type IB catalyzing the interconversion between different topological states of DNA (Brill *et al*. 1987). Lack of either *TOP1* or *TOP2*, encoding topoisomerase type II that also relaxes the helical tension of intracellular DNA, does not affect replication, transcription, and mRNA synthesis (Salceda *et al*. 2006, Bermejo *et al*. 2007). Top1p has the risk to form DNA–Protein Crosslinks (DPCs), a specific type of DNA lesions caused by Top1p being covalently and irreversibly bound to DNA (Fielden *et al*. 2018, El Dika 2020). A previous study showed that *TOP1* deletion improved the gene expression of telomere-proximal regions that are enriched for stress-activated genes (Lotito *et al*. 2008). Furthermore, *TOP1* deletion also increased the resistance to reactive oxygen species (ROS)-induced oxidative stress (Litwin *et al*. 2013, Terzioğlu *et al*. 2020). Protein production is often associated with increased oxidative stress and therefore deletion of *TOP1* might be beneficial here.

Ede1p is a crucial scaffold protein for defining the early stages of endocytosis and is one of the earliest-arriving proteins in this process. This protein recruits other early-phase proteins to the nascent clathrin-mediate endocytosis (CME) site and then promotes site initiation, selection, and maturation (Boeke *et al*. 2014). Previous studies showed that the deletion of *EDE1* impaired endocytosis by causing fewer CME site initiations and reducing the lifetimes of other endocytic proteins, such as Sla1p, required for cortical actin cytoskeleton assembly (Kaksonen *et al*. 2005, Stimpson *et al*. 2009), which might decrease endocytosis of the secreted α-amylase and, therefore, enhance protein production. Ede1p is a large, multimodular domain protein, consisting of three N-terminal Eps15 homology (EH) domains, followed by a proline-rich region and a central-coiled region, and a C-terminal ubiquitin-associated (UBA) domain (Lu and Drubin 2017). As no obvious difference in the localization of Ede1p to the endocytic site was observed in the C-terminal and UBA domain deleted strain (*ede1^∆901^^–^^1381^*; Boeke *et al*. 2014), it supports our result that the amino acid change of *EDE1*(E1057K) did not affect α-amylase production due to the mutation being located in a domain presumably noncritical for the function of *EDE1*.

Gos1p, located to the medial Golgi, is a vesicle-soluble N-ethylmaleimide-sensitive factor attachment protein receptor (v-SNARE) protein (Beznoussenko *et al*. 2016), which is involved in vesicle transport within the Golgi complex (McNew *et al*. 1998). Previously, a *GOS1* deletion strain resulted in hypersecretion of ER-resident proteins, which potentially reflected a defect in retrograde directed vesicle transport (McNew *et al*. 1998). Furthermore, the disruptions of Gos1p improved heterologous amylase production and the cell growth was only slightly reduced which might be due to partial deletion of *GOS1* (Huang *et al*. 2018). The point mutation of *GOS1* might also have resulted in reduced retrograde transport in the Golgi, thus benefitting α-amylase secretion. Moreover, previous studies demonstrated that balancing the bidirectional vesicular trafficking pathway involved in protein transportation between the ER and the Golgi enhanced heterologous protein secretion (Bao *et al*. 2017, 2018). Both engineering anterograde trafficking by moderately overexpressing *SEC16* and engineering retrograde trafficking by overexpressing *GLO3* increased the secretion of α-amylase, endoglucanase I from *Trichoderma reesei* and glucan-1,4-α-glucosidase (GLA) from *Rhizopus oryzae* (Bao *et al*. 2017, 2018). Similarly, protein vesicular trafficking has been engineered in *Komagataella phaffii* as well (Montegna *et al*. 2012, Bharucha *et al*. 2013). It thus is an important strategy for efficient protein production to engineer the anterograde and retrograde vesicular trafficking between ER and the Golgi or within the Golgi complex.

Mag2p represents a cytoplasmic protein of so far unknown function, although it is predicted to contribute to the repair of alkylated DNA damage (Samanta and Liang 2003, Iwahashi *et al*. 2006).

### The modification of seven genes in subset 3 has a positive effect on α-amylase production

**Figure 5.**
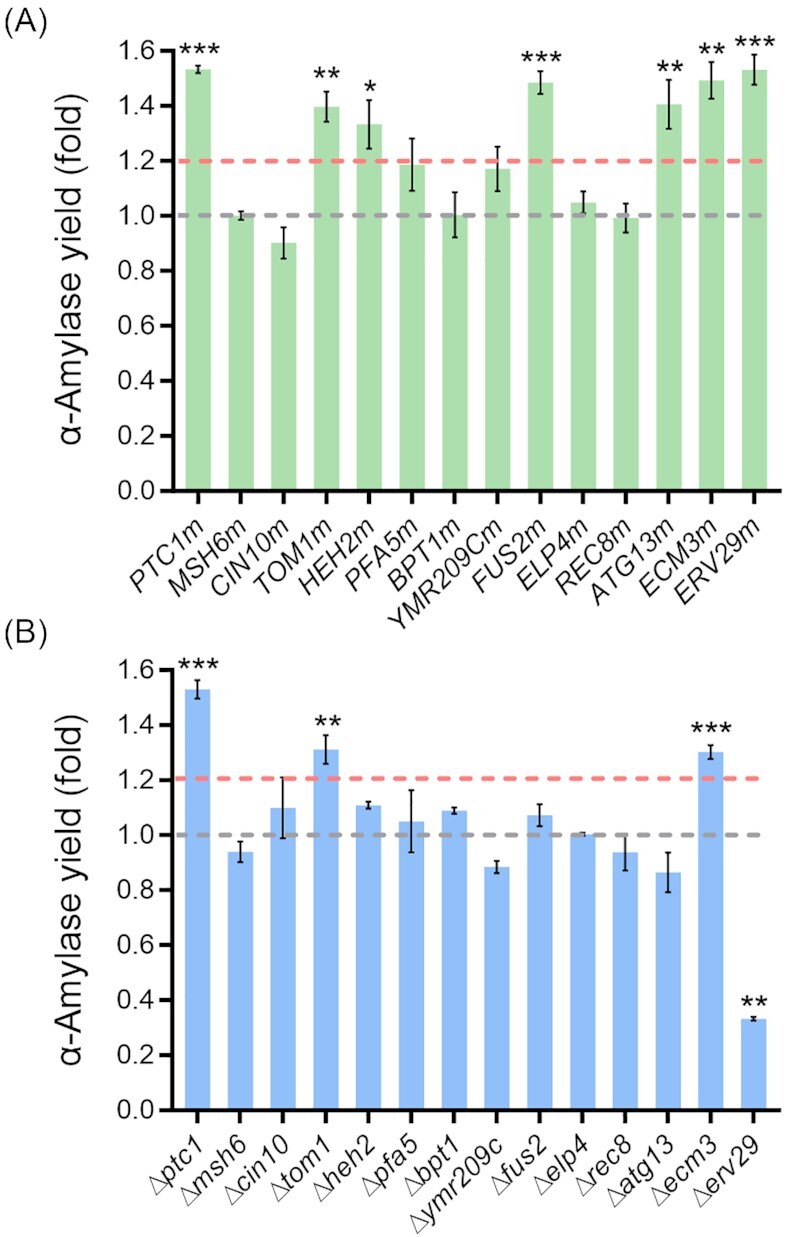
α-Amylase production after introduction of point mutations (A) or gene deletions (B) of genes identified as additionally mutated in strain B184 (subset 3). Supernatants were collected after 96 h of cultivation in SD-2xSCAA medium at 30°C and the parental strains K01 or KC01 were used as controls. The dotted gray line indicates the α-amylase production of the control strain for comparison. The dotted pink line indicates a 1.2-fold increase in α-amylase production compared to the control strain. The *Y*-axis represents the fold change of α-amylase activity per OD_600_ (yield) compared to the control strain. Data shown are average values ± SD of independent biological triplicates. The statistical significance was listed when the α-amylase yield change was more than 1.2-fold or less than 0.8-fold compared to the control (*P < .05, **P < .01, and ***P < .001).

Finally, we tested 15 gene targets from subset 3. Similar to *USO1*, as we were unable to obtain the mutated allele nor the deletion for *CWC2* (Lu *et al*. 2012), 14 gene targets were ultimately tested. A total of seven out of the 14 point mutations significantly increased the α-amylase yield ( *P* < .05) compared to the control strain (Fig. 5A). A total of six out of the 14 single gene deletions significantly changed the α-amylase yield, with four increasing α-amylase production and two decreasing α-amylase production (*P* < .05) compared to the control strain (Fig. 5B). Figure S6 (Supporting Information) illustrates the biomass changes compared with the control strain. Seven genes were further investigated due to the significant improvement in α-amylase production by more than 1.2-fold (Fig. 5). Three genes, *PTC1*, *TOM1*, and *ECM3*, improved protein production for both the point mutation and gene deletion. For *HEH2*, *FUS2*, *ATG13*, and *ERV29*, only the point mutation significantly increased α-amylase production by more than 1.2-fold, while the deletion of *ERV29* significantly decreased protein production (*P* < .01).

Ptc1p, a protein of the type 2C protein phosphatase (PP2C) family, regulates signal transduction by dephosphorylating Hog1p and inactivating the mitogen-activated protein kinase (MAPK) cascade involved in osmolarity sensing (Ariño *et al*. 2011). In response to hyperosmotic stress, cells rapidly stimulate the high osmolarity glycerol (HOG) pathway, which leads to phosphorylation and activation of MAP kinase Hog1p and, therefore, promotes its nuclear import (Ferrigno *et al*. 1998, Ariño *et al*. 2011). To restore the osmotic balance, the nuclear-localized Hog1p upregulates about 600 genes through phosphorylating osmoresponsive transcription factors (Hohmann 2002). More intracellular resources are allocated to produce various small molecules, such as glycerol (Westfall *et al*. 2004). A previous study showed deletion of *PTC1* enhanced the activity of the HOG pathway and resulted in an increase of the glycerol content (Jiang *et al*. 1995), and therefore, helps relieve cellular osmotic stress. Consistently, we found that the *PTC1* deletion strain and point mutation strain both produced more glycerol compared to the control strain in our study (Figure S7A, Supporting Information). The accumulation of intracellular glycerol has been proved to be benificial for heterologous protein production when yeast cells are exposed to a hypertonic medium (Shi *et al*. 2003, Chen *et al*. 2021). On the other hand, the deletion of *PTC1* reduces the cell wall β-1,6-glucan levels by activating Exg1p (Jiang *et al*. 1995), an exo-β-glucanase of the cell wall (Cappellaro *et al*. 1998). We also observed a reduced growth rate (0.14 in the *PTC1* deletion strain vs. 0.25 in the control strain) and lower final biomass (0.42-fold) in the *PTC1* deletion strain (Figures S6B and S7B, Supporting Information). However, the cell growth and final biomass were not affected in the *PTC1* mutation strain (Figures S6A and S7B, Supporting Information), and the glycerol content in the supernatant of the *PTC1* mutation strain was higher than in that of the control strain but lower than in that the supernatant of the *PTC1* deletion strain after 40 h of shake flask fermentation (Figure S7A, Supporting Information). Furthermore, the amino acid substitution of Y144F is located within the catalytic domain (10–279) of Ptc1p (Ariño *et al*. 2011). All of these indications led us to speculate that weakening the activity of Ptc1p rather than deletion of *PTC1* might be beneficial for α-amylase secretion. This is also consistent with a previous study, where a moderate level of osmolarity was the most beneficial strategy for enhancing the secretion of heterologous proteins in *Yarrowia lipolytica* cultures (Kubiak *et al*. 2019).

Tom1p, an E3 ubiquitin ligase, is involved in mRNA export from the nucleus to the cytoplasm and the regulation of various transcriptional coactivators (Niño *et al*. 2013, Niekamp *et al*. 2019). Based on the literature, Yra1p, the first characterized nonshutting mRNA export adaptor, forms a tripartite complex Nab2p-Mex67p-Yra1p on the released mRNA ribonucleoprotein particles (mRNPs), which helps the mRNP exit through nuclear pores (Iglesias *et al*. 2010). Subsequently, Yra1p dissociates from the mRNP before its export from the nucleus after ubiquitination mainly by Tom1p (Infantino *et al*. 2019, Infantino and Stutz 2020), which ensures the export of only mature mRNPs (Iglesias *et al*. 2010). Deletion of *TOM1* abolishes ubiquitin ligase activity and results in the accumulation of mRNA near the nuclear pore complex, and thus decreases efficient mRNA export (Duncan *et al*. 2000). α-Amylase is a rather large and complex protein and the CPOTud plasmid expression system generates high amounts of recombinant protein, which results in misfolding stress and more cellular resources being invested in protein production and degradation (Liu *et al*. 2012). In the AAC strain, the global transcription and translation was downregulated and the stress response, UPR and ER processing were upregulated upon amylase expression (Liu *et al*. 2012). Besides, several stress-related genes, such as *HOG1* and *YAP1*, were significantly upregulated (Liu *et al*. 2012). This indicates that α-amylase production causes a protein folding burden, and therefore, leads to cellular oxidative stress. Indeed, all the strains in this evolutionary line showed higher ER stress compared to the non-α-amylase-producing strain, which suggested the ER stress results from α-amylase production (Huang *et al*. 2017). In addition, the ROS level was lower in B184 than in AAC (Huang *et al*. 2017). We speculate that the reduction of the mRNA export and the peptides translocated to the ER will potentially contribute to a decrease in ROS generation by protein folding stress, which allows cells to allocate more resources to protein secretion, and therefore, increases efficient heterologous protein production. Furthermore, lack of *TOM1* would cause the accumulation of ribosomal proteins (Sung *et al*. 2016). Overexpression of ribosomal proteins in *S. cerevisiae*, such as *RPP0*, which is essential for large ribosomal subunit assembly, improved heterologous protein secretion (Wentz and Shusta 2008). However, deletion of some ribosomal protein genes could also enhance heterologous protein production by increasing cotranslational folding or inhibiting vesicle transport (Kitagawa *et al*. 2011, Liao *et al*. 2019). As the biosynthesis of ribosomal proteins has a connection with heterologous protein production, this could represent another potential reason for the effect of the *TOM1* deletion. The *TOM1*(S3218F) substitution occurred at the hect (Homologous to the E6-AP Carboxyl Terminus)-domain (Duncan *et al*. 2000), a common domain in a specific subclass of ubiquitin–protein ligases, which might impair the ability of Tom1p to accept ubiquitin from ubiquitin-conjugating enzyme and thus reduce the enzymatic activity of Tom1p (Huibregtse *et al*. 1995, Duncan *et al*. 2000). The presumably reduced activity of Tom1p(S3218F) led to a result in protein production similar to the *TOM1* deletion.

Another validated target, Atg13p, consisting of an N-terminal HORMA (from Hop1p, Rev7p, and Mad2p) domain (residues 2–268) and a C-terminal intrinsically disordered region (residues 269–738), is a central regulatory factor, which acts on the initiation of preautophagosomal structure (PAS) assembly and consequently the formation of autophagosomes (Suzuki and Ohsumi 2007, Popelka and Klionsky 2017). During the nucleation of autophagosome formation, Atg9p, an integral membrane protein essential for phagophore membrane formation (Suzuki *et al*. 2015), and Atg14p, an autophagy-specific subunit of the phosphatidylinositol 3-kinase complex I (Jao *et al*. 2013), are recruited to the PAS. The recruitment occurs through interaction with the N-terminal HORMA domain of Atg13p, demonstrating the importance of Atg13p for autophagy (Jao *et al*. 2013, Suzuki *et al*. 2015). Under various external stresses, such as hypoxic, osmotic, metabolic, and starvation stress, autophagy is dramatically activated to help in stress adaptation and recycle cytosolic macromolecular components such as proteins and organelles (Wang *et al*. 2015, Farré and Subramani 2016), which plays an important role in maintaining cellular homeostasis. In contrast to the *ATG13* point mutation, deletion of *ATG13* resulted in a slightly lower protein secretion than the control strain. Previously, deletion of *ATG13* decreased autophagy and cell viability under starvation conditions (Funakoshi *et al*. 1997). Indeed, a reduced final biomass in the *ATG13* deletion strain was observed compared to the control strain (14% reduction; Figure S6B, Supporting Information), while the point mutation of *ATG13* did not affect biomass production (Figure S6A, Supporting Information). According to other studies, premature mutation of Mtc6p, a hypothetical ER protein, attenuated autophagy, and thus dramatically increased the secretory expression of heterologous proteins, including ruminal feruloyl esterase (Est1E), mannase (Man330), β-1,4-endoxylanase (XynCDBFV), and enhanced green fluorescent protein (EGFP), in *Kluyveromyces marxianus* (Liu *et al*. 2018). Reduced expressed levels of several autophagy-related genes enhanced the production of heterologous bovine chymosin (CHY) in *Aspergillus oryzae* (Yoon *et al*. 2013). In response to cell stress, the substitution of *ATG13*(L152F) situated within the HORMA domain might affect the interaction of Atg13p with Atg9p and Atg14p, which are required in the nucleation process. This potentially decreased autophagosome formation, which might have been beneficial for amylase production. On the other hand, by adding 3 µM autophagy‐inducing peptide in the serum‐free cultures, IgG concentration significantly improved by over 2-fold compared to the control cultures in Chinese Hamster Ovary (CHO) cells (Braasch *et al*. 2021). Treatment with the appropriate autophagy inducers such as rapamycin and everolimus increased the production of secreted antibody scFv-Fc in insect cells (Nakanuma *et al*. 2020). Mounting evidence supports that it is a promising approach to enhance recombinant protein production by modulation of the autophagy pathway. Also, the identified target *EDE1* (see above) is related to autophagy, which could further emphasize the importance of regulating the autophagic process.

Fus2p is a key regulator of cell fusion and quickly activated by mating pheromones in a highly regulated manner (Kim and Rose 2012). It contains a Dbl homology domain with Rho guanine exchange factor proteins (GEFs), which play critical roles in yeast mitosis and cell cycle progression (Ydenberg and Rose 2009). We, thus speculate that Fus2p directly or indirectly affects the cell cycle in yeast. In connection with this, a previous study demonstrated that mitotic cells block pheromone signaling, allowing Fus2p to remain in the nucleus to ensure proper cell cycle progression (Kim and Rose 2012). The youngest daughter cells (newborn G1 phase cells) do not secrete detectable amounts of recombinant human cytokine in *S. cerevisiae* into the medium until they reach cell division (Frykman and Srienc 2001), showing that the cell cycle plays a vital role in protein secretion. Furthermore, a previous study indicated that the specific production rate of rice α-amylase in *S. cerevisiae* reached its maximum during the M phase (Uchiyama *et al*. 1997), likewise suggesting a cell cycle dependency of protein secretion. Several genes (*ERV25*, *EMP24*, *SEC28*, *SLY41*, *UFE1*, *GYP6*, and *SSO2*), involved in various secretory processes such as ER and Golgi trafficking, vesicle coating, and fusion of secretory vesicles to the plasma membrane, are expressed in a cell cycle-dependent pattern (Spellman *et al*. 1998). According to GO Slim Term analysis, *TOP1*, *EDE1*, and *TOM1* are associated with the regulation of the cell cycle (Table S1, Supporting Information), which implies that the cell cycle might be an interesting target for optimizing the production of secreted protein.

Erv29p is a transmembrane receptor cycling between ER and the Golgi apparatus, which is required for packaging glycosylated pro-α-factor (gpαf) into COPII vesicles for anterograde transport (Foley *et al*. 2007, Casler *et al*. 2020). In this work, the secretion of α-amylase was guided by the pre-pro-α-factor signal peptide. Our result showed that *ERV29* deletion reduced the protein secretion by 67% (Fig. 5B), which is consistent with previous reports that lack of Erv29p led to a reduced rate of gpαf transportation and the intracellular accumulation of protein in the ER (Belden and Barlowe 2001, Besada-Lombana and Da Silva 2019). Conversely, overexpression of *ERV29* was previously shown to enhance the secretion of α-amylase with a pre-pro-α-factor leader (Huang *et al*. 2018) and heterologous endoglucanase CelA with an Ost1-pro-α-factor hybrid leader (Besada-Lombana and Da Silva 2019), respectively. Furthermore, another study indicated that the abundance of Erv29p is decreased at a dilution rate of 0.2/h in chemostat culture by 9.6-fold in B184 compared to AAC (Qi *et al*. 2020), but our result demonstrated that the point mutation of *ERV29* significantly increased α-amylase secretion by 1.53-fold (Fig. 5A). The decrease in protein abundance and increase in α-amylase secretion have led us to speculate that the amino acid substitution of *ERV29*(L193S) potentially increased the affinity of Erv29p to the pro-α-factor and therefore enhanced the Erv29p-dependent export.

Heh2p is an inner nuclear member protein and is involved in nuclear location signaling and Ecm3p is involved in signal transduction and genotoxic response. Their detailed biological functions are however still unknown.

To test the capacities of the targets with the improved α-amylase production for the production of other proteins, we evaluated production of two additional heterologous proteins, GLA from *R. oryzae* and Nanobody (Nan, antibody single V-type domain) from the Camelidae family, in five of our engineered strains. Specifically, we chose one point mutation strain and one gene deletion strain with the highest α-amylase production in the respective subset. *CBP2m*, *TIR3m*, *∆top1*, *PTC1 m*, and *∆tom1* were selected since no deletion strain in subset 1 increased α-amylase production. Among them, *TIR3m*GLA and *∆top1*GLA showed a significant increase in GLA secretion and *∆top1*Nan was beneficial for Nan secretion (Figure S8, Supporting Information). The results indicate that at least some of the targets are suitable candidates for increasing protein production in general, even though not all targets have the same effect on all recombinant proteins. This is also reflected by the fact that strain B184 combining all point mutations increased the production of most recombinant proteins tested albeit at different extents (Huang *et al*. 2015, 2017, Wang *et al*. 2021).

## Conclusion

**Figure 6.**
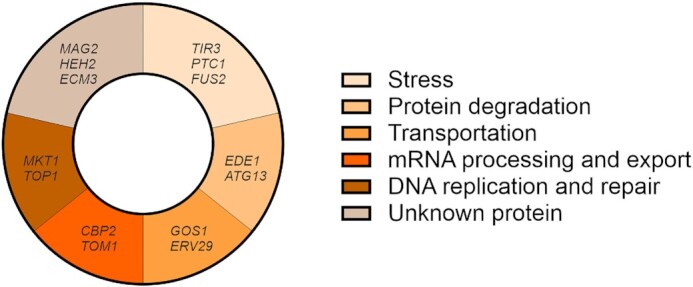
The enriched bioprocesses of 14 genes involved in the improvement of α-amylase secretion using GO Slim Process categories.

To summarize, we presented three CRISPR/Cas9-based strategies to introduce nucleotide substitutions in this work. These strategies are universally applicable dependent on the location of the point mutation in relation to the nearest PAM site. Based on the evaluation of all protein-altering mutations along the evolutionary path toward B184, 14 point mutations and/or deletions were shown to be beneficial for α-amylase secretion. For seven genes, only the point mutations had a positive effect; in four cases, both point mutation and gene deletion increased α-amylase secretion; and in three cases, only the gene deletion was beneficial. This shows that a high proportion of the mutations acquired during the evolution process contributed to the improved phenotype and also demonstrates that it is important to verify their effect by reintroducing the point mutation instead of simply deleting or overexpressing the mutated genes. Out of the validated genes, 11 targets were newly identified in this work. These genes are related to various stress-related processes, protein degradation, transportation, mRNA processing and export, DNA replication, and repair (Fig. 6; Table S1, Supporting Information). It is, therefore, very important to balance these intracellular processes to improve protein secretion. Detailed exploration of the functions and effects of protein-altering mutations will help to understand the mechanisms involved in protein secretion in *S. cerevisiae*. The gene targets identified here will also provide guidelines for designing other efficient cell factories for protein production.

## Materials and methods

### Strains

*Escherichia coli* DH5α was used for plasmid construction and amplification. The *S. cerevisiae* strain K01 (*MAT*a *URA3 HIS3 LEU2 TRP1 SUC2 MAL2-8^c^ tpi1(41–707)::loxP* with pAlphaAmyCPOT) was used as the parental strain in this study. K01 was derived from CEN.PK 530–1CK carrying the plasmid pAlphaAmyCPOT, containing a *TPI1* promoter, a yeast α-factor leader and an α-amylase gene from *A. oryzae* (Liu *et al*. 2012). The only difference between K01 and AAC is that the genomically integrated *KanMX4* marker has been removed from K01. All mutated genes in this study are listed in Table S1 (Supporting Information). All *S. cerevisiae* strains used are listed in Table S2 (Supporting Information).

### Culture conditions

*Escherichia coli* cells were grown in LB medium (10 g/l peptone from casein (Merck), 10 g/l NaCl (Merck), and 5 g/l yeast extract (Merck)) with 100 mg/l ampicillin (Sigma) overnight at 37°C and 180 rpm on a rotary shaker. Yeast strains were regularly grown in YPD medium, consisting of 20 g/l peptone from meat (Merck), 10 g/l yeast extract, and 20 g/l glucose (Sigma). Yeast strains containing *KanMX*-based plasmids were selected in YPD + G418 medium, consisting of YPD medium with 200 mg/l G418 (Sigma). Yeast strains without plasmids were grown in YPE medium, consisting of 20 g/l peptone from meat, 10 g/l yeast extract, 10 g/l ethanol (VWR), and 0.5 g/l glucose. Starch agar plates consisted of 10 g/l starch, 6.9 g/l yeast nitrogen base without amino acids (Formedium), 0.04 g/l glucose, and 20 g/l agar (Merck). Starch and ethanol agar plates consisted of 10 g/l starch, 6.9 g/l yeast nitrogen base without amino acids, 10 g/l ethanol, 0.04 g/l glucose, 790 mg/l complete supplement mixture (Formedium), and 20 g/l agar. A total of 20 g/l agar was added for making all plates. All yeast cells were grown at 30°C in this study.

Shake flask batch fermentations for the measurement of α-amylase production were performed in SD-2xSCAA medium (pH 6.0) (Wittrup and Benig 1994), which consisted of 6.9 g/l yeast nitrogen base without amino acids, 5.4 g/l Na_2_HPO_4_, 8.56 g/l NaH_2_PO_4_·H_2_O, 1 g/l BSA, 20 g/l glucose, 0.190 g/l Arg, 0.400 g/l Asp, 1.260 g/l Glu, 0.130 g/l Gly, 0.140 g/l His, 0.290 g/l Ile, 0.400 g/l Leu, 0.440 g/l Lys, 0.108 g/l Met, 0.200 g/l Phe, 0.220 g/l Thr, 0.040 g/l Trp, 0.052 g/l Tyr, and 0.380 g/l Val. A single colony was used to inoculate 2 ml of SD-2xSCAA medium in a 14-ml tube, which was then incubated at 200 rpm for 24 h. Main cultures were 10 ml of SD-2xSCAA medium in 50-ml unbaffled narrow neck shake flasks inoculated with the precultures at an initial optical density at 600 nm (OD_600_) of 0.05. These were incubated for 96 h at 200 rpm orbital shaking if not specified otherwise. For oxygen-limited cultivation, main cultures were grown in 20 ml of SD-2xSCAA medium in 100-ml unbaffled shake flasks sealed with rubber stoppers inoculated to an initial OD_600_ of 0.05 for 96 h at 160 rpm orbital shaking. To minimize oxygen intake, the rubber stopper was equipped with a U-shaped glass tube filled with sterile water. For measuring cell growth, main cultures were grown in 250 µl of SD-2xSCAA medium in a 96-well microplate inoculated with the precultures at an initial OD_600_ of 0.05 for 96 h at 250 rpm orbital shaking in the growth profiler 960 (EnzyScreen). OD_600_ values (referred to here as OD_600_ equivalents) were recorded at intervals of 30 min.

### Plasmid construction

Guide RNA (gRNA) expressing plasmids were constructed based on all-in-one plasmid pECAS9-gRNA-kanMX (Zhu *et al*. 2017). This plasmid with a *kanMX* marker was used for the expression of both guide RNA and the high-fidelity *cas9* nuclease gene. The specific gRNA sequences were designed using the free online tool CRISPRdirect (https://crispr.dbcls.jp). The plasmid pECAS9-gRNA-kanMX as a backbone was divided into two fragments, which were amplified using primers pECAS-gRNA-F/pECAS-KanMX-R and pECAS-TEF1-F/pECAS-gRNA-R. The new gRNA plasmid was generated through assembling the gRNA sequence-containing DNA fragment and the two backbone fragments by *in vitro* Gibson assembly (NEB). A standard protocol was used for *E. coli* transformations (Inoue *et al*. 1990). All plasmids were verified using sequencing by Eurofins Genomics. Plasmids used are listed in Table S3 (Supporting Information). Primers used are listed in Table S4 (Supporting Information).

### Genetic manipulation

Genomic modification by the CRISPR/Cas9 system was carried out by cotransforming the background strain with both a gRNA expressing plasmid and a repair fragment. In order to improve the cleavage efficiency of Cas9 nuclease, a Cas9 expression cassette (*TEF1p*-*cas9*-*CYC1t*) under control of the strong and constitutive *TEF1* promoter was integrated at the chromosome XI-3 locus in strain K01 using the gRNA plasmid pECAS9-gRNA-XI-3. This Cas9 cassette was amplified from the plasmid pECAS9-gRNA-kanMX using primer pairs TEF1p-XI-3-up-F/XI-3-dn-CYC1t-R. The upstream and downstream homologous arms were amplified from IMX581 genomic DNA using primer pairs XI-3-up-F/XI-3-up-R and XI-3-dn-F/XI-3-dn-R, respectively. The repair fragment was assembled by fusing these three DNA fragments using PCR. A total of 500 ng of pECAS9-gRNA-XI-3 and 300 ng of the repair fragment were cotransformed into K01, resulting in KC01. K01 and KC01 were used as the background strains for introducing nucleotide point mutations. In this study, point mutations were introduced into the chromosome adopting three strategies according to their locations (Fig. 2).

Scenario I: the mutated site is located within the gRNA recognition sequence (20 bp). In this situation, the point mutation or gene deletion was performed directly by cotransformation of the repair fragment and the gRNA expressing plasmid. The 100-bp repair fragment containing the mutation was amplified by using primer pairs containing a 20-bp overlap. The forward primer consisted of a 40-bp upstream homologous arm and the 20-bp mutated gRNA recognition sequence. The reverse primer consisted of a 40-bp downstream homologous arm and the 20-bp mutated gRNA recognition sequence. For the gene deletion, the 80-bp repair fragment consisted of a 40-bp upstream homologous arm and a 40-bp downstream homologous arm of the respective target gene and was generated by annealing two complementary 80-bp primers.

Scenario II: the gRNA selected for scenario I does not result in correct transformants or the mutated site is not located within the gRNA recognition sequence and the gene is essential. Here, a gRNA target site close to the desired mutation was chosen and one or two bases of PAM sequence were changed without changing the resulting amino acid sequence while introducing the repair fragment. The repair fragment for introducing the gene mutation was amplified by using primer pairs containing a 20-bp overlap. Taking the example of the PAM sequence upstream of the target mutation site, the forward primer consisted of a 40-bp homologous arm upstream of the PAM sequence and the sequence from the mutated PAM site to the site to be mutated. The reverse primer consisted of a 40-bp homologous arm downstream of the mutated site and the sequence from the mutated site to the mutated PAM site.

Scenario III: the gRNA selected for scenario I does not lead to correct transformants or the mutated site is not located within the gRNA recognition sequence and the gene is nonessential. In this situation, a two-step approach was carried out. First, the gene was deleted while a heterologous gRNA recognition sequence was introduced to repair the genome DSB. The repair fragment for deletion bridged a 40-bp upstream homologous arm and 40-bp downstream homologous arm of the target gene using a heterologous gRNA recognition sequence and its PAM sequence. In this study, the PsTAL1 sequence, containing heterologous gRNA recognition sequence (GCATCCAAAACTTGTGGAAC), its PAM sequence (TGG) and additional three nucleotides next to the PAM sequence (TTC), were selected from the *TAL1* gene of *Pichia stipitis*. The ca. 100 bp repair fragment was amplified using a pair of primers containing this 26 bp overlap. Cotransformation of gRNA encoding plasmid and repair fragment resulted in a strain with heterologous gRNA recognition sequence. In the second step, the introduced PsTAL1 sequence was replaced with the mutated gene fragment. The gRNA-expressing plasmid was gRNA-PsTAL1 targeting the introduced gRNA recognition site. The repair fragment containing the desired point mutation was assembled by fusing several DNA fragments which had 35–40 bp overlap between the fragments. Taking single-nucleotide mutation as an example, two fragments were amplified from IMX581 genomic DNA by using primer pairs Gene-up/Gene-mut-R and Gene-mut-F/Gene-dn, respectively. The mutated nucleotide was included in the primers Gene-mut-F/Gene-mut-R.

For the construction of the mutation and deletion strains, 500 ng of the respective gRNA containing plasmid and 300 ng of the repair fragment were cotransformed into the background strain according to the standard lithium acetate method (Gietz and Schiestl 2007). Transformants were selected on YPD + G418 plates. For deletion strains, transformants were verified by colony PCR using the SapphireAmp® Fast PCR Master Mix (TaKaRa). For mutation strains, transformants were verified by PCR and Sanger sequencing of the PCR product. Three independent clones with the correct gene modification were cultured in nonselective YPD medium for 24 h to remove the corresponding gRNA plasmids and recycle the *KanMX* marker. Then, these three clones were streaked onto YPD plates to obtain single colonies. Subsequently, to confirm the loss of gRNA plasmid, single colonies were streaked on both YPD + G418 and YPD plates, respectively. The colonies that were not able to grow on YPD + G418 plates but YPD plates were picked and used for shake flask batch fermentations.

### Plasmid loss

For the production of other heterologous proteins, GLA and Nan, the α-amylase plasmids were first eliminated from the *CBP2m*, *TIR3m*, *∆top1*, *PTC1m*, *and ∆tom1* strains by continuous selective transfers in YPE medium. Briefly, a single colony was used to inoculate 1.5 ml of YPE medium. Then, 10 μl of the culture were transferred to 1.5 ml of fresh YPE medium twice, and subsequently streaked on a YPE plate. To confirm the loss of α-amylase expression plasmid, single colonies were spotted on both a starch agar plate and a starch and ethanol agar plate, respectively. The colonies that were not able to grow and form a halo on the starch agar plate but were able to grow on the starch and ethanol agar plate without halo formation were picked and used for transformation. Subsequently, the plasmids pCP-αGLA and pCP-Nan were used to transform the parental strain and plasmid-depleted modified strains, respectively.

### Protein quantification

A volume of 1 ml of cell cultures from shake flasks was centrifuged for 4 min at 3000 *g* to collect the supernatant. The concentration of secreted α-amylase and glucan 1,4-α-glucosidase was determined by measuring enzyme activity in the culture supernatant. An α-amylase assay kit (Megazyme) was used to measure α-amylase activity and the commercial α-amylase from *A. oryzae* (Sigma) was used as a standard. The mass of α-amylase was converted from its activity using 69.6 U/mg as the conversion coefficient (Liu *et al*. 2012). An amyloglucosidase assay kit (Megazyme) was used to measure the activity of glucan 1,4-α-glucosidase.

The protein amount of Nan was determined by western blot analysis as described previously (Wang *et al*. 2021). Briefly, the supernatants were diluted 1:1 in 2x loading dye supplemented with 10% 2-mercaptoethanol, and heated for 5 min at 98°C. Then, 10-µl samples were loaded on 4%–20% Mini-PROTEAN® TGX Stain-Free^TM^ Protein Gels (Bio-Rad). Following electrophoresis, protein bands were transferred to 0.2 µm polyvinylidene fluoride membranes (Bio-Rad). After blocking with 5% nonfat milk, the membranes were probed with anti-6x-His-tag monoclonal antibody (Thermo Scientific; 1:2000 dilution) and subsequently polyclonal goat antimouse immunoglobulin antibody conjugated with horseradish peroxidase (HRP; Dako; 1:1000 dilution). After the membranes had been treated with HRP substrate—SuperSignal West Dura Extended Duration Substrate (ThermoFisher Scientific), protein bands were visualized by a ChemiDoc XRS image analyzer (Bio-Rad). The protein intensity was analyzed by the software Image Lab 6.0.1 and the protein amount was estimated. The statistical significance of the results from independent triplicates was assessed using a two-tailed Student’s *t*-test.

### Metabolite quantification

A volume of 1 ml of cell cultures was centrifuged for 10 min at 13 000 rpm and the supernatant was filtered using a 0.45-μm syringe filter. Glycerol concentration was measured by an Ultimate 3000 HPLC (high-performance liquid chromatography) equipped with an Aminex HPX-87 G column (Bio-Rad). The column was eluted for 30 min at 45°C with 5 mM H_2_SO_4_ at a flow rate of 0.6 ml/min.
